# Glucose Metabolism-Modifying Natural Materials for Potential Feed Additive Development

**DOI:** 10.3390/pharmaceutics16091208

**Published:** 2024-09-13

**Authors:** Wei-Chih Lin, Boon-Chin Hoe, Xianming Li, Daizheng Lian, Xiaowei Zeng

**Affiliations:** 1 School of Pharmaceutical Sciences (Shenzhen), Shenzhen Campus of Sun Yat-sen University, Shenzhen 518107, China; weichih.lin@kemin.com (W.-C.L.); boonchin.hoe@kemin.com (B.-C.H.); 2 Kemin (China) Technologies Co., Ltd., Zhuhai 519040, China; 3 Shenzhen People’s Hospital (The Second Clinical Medical College, Jinan University, The First Affiliated Hospital, Southern University of Science and Technology), Shenzhen 518020, China; lixianming1828@hotmail.com

**Keywords:** glucose metabolism, phytochemicals, probiotics, prebiotics, chromium, natural feed additives, antioxidants, glucose transporters

## Abstract

Glucose, a primary energy source derived from animals’ feed ration, is crucial for their growth, production performance, and health. However, challenges such as metabolic stress, oxidative stress, inflammation, and gut microbiota disruption during animal production practices can potentially impair animal glucose metabolism pathways. Phytochemicals, probiotics, prebiotics, and trace minerals are known to change the molecular pathway of insulin-dependent glucose metabolism and improve glucose uptake in rodent and cell models. These compounds, commonly used as animal feed additives, have been well studied for their ability to promote various aspects of growth and health. However, their specific effects on glucose uptake modulation have not been thoroughly explored. This article focuses on glucose metabolism is on discovering alternative non-pharmacological treatments for diabetes in humans, which could have significant implications for developing feed additives that enhance animal performance by promoting insulin-dependent glucose metabolism. This article also aims to provide information about natural materials that impact glucose uptake and to explore their potential use as non-antibiotic feed additives to promote animal health and production. Further exploration of this topic and the materials involved could provide a basis for new product development and innovation in animal nutrition.

## 1. Introduction

Glucose metabolism is fundamental to maintaining energy balance and overall health in both humans and animals. It involves a complex set of biochemical processes that convert glucose into usable energy, which is crucial for the proper functioning of cells and organs. In the context of livestock, efficient glucose metabolism is particularly important as it directly impacts growth, productivity, and health. Optimizing glucose metabolism can lead to improved feed conversion rates, better growth performance, and enhanced resistance to metabolic disorders and stress. Therefore, it is essential to maintain proper glucose homeostasis in farm animals, especially in high-producing ones, such as cattle, broilers, and swine, which have high energy demands and are prone to metabolic stress [1,2]. Consequently, this area could become a trending topic for researchers and industry professionals seeking to enhance livestock production systems’ metabolic efficiency and sustainability. By targeting glucose metabolism, it is possible to develop innovative nutritional strategies that support the health and productivity of livestock, aligning with broader goals of sustainable and efficient agricultural practices.

The rising demand for sustainable agriculture is driven by the growing need for efficient farming practices that minimize environmental impact and ensure long-term viability. As global populations increase, there is an urgent requirement to produce more food with fewer resources, pushing the agricultural sector towards innovative solutions that enhance productivity while preserving ecological balance [3]. Concurrently, consumers are becoming more discerning, with a marked preference for natural and organic products perceived as healthier and more environmentally friendly. This shift in consumer behavior is compelling producers to adopt practices that not only boost efficiency but also align with the principles of sustainability and natural product use [4]. Consequently, the integration of natural materials as feed additives is gaining traction as a strategy to meet these dual demands, promoting both the health of livestock and the sustainability of agricultural systems [5].

A diverse array of natural materials known for their potential to modulate glucose metabolism is available, focusing on plant extracts, phytochemicals, probiotics, and prebiotics. Plant extracts, derived from various herbs, fruits, and vegetables, are rich in bioactive compounds that can enhance metabolic processes. These extracts often contain polyphenols, flavonoids, and terpenoid essential oils, which have been shown to improve insulin sensitivity and glucose uptake, thereby promoting better energy balance. Furthermore, phytochemicals can also be extracted from food industry byproducts, such as grape pulp, citrus peel, olive pomace, and apple pomace, which are usually discarded or used as low-value animal feed. These byproducts are rich in phytochemicals, especially polyphenols and flavonoids, and can be used as alternative or complementary sources of functional feed additives for farm animals. Using these byproducts, the food industry can reduce its environmental impact and waste generation, and the animal industry can reduce its feed costs and increase its profitability. Therefore, using phytochemicals as functional feed additives for farm animals can be considered a sustainable choice that can improve animal farming practices and the quality of animal products [6,7]. Probiotics are live microorganisms that can benefit the host animal by modulating its intestinal microbiota. At the same time, prebiotics are non-digestible carbohydrates that can selectively stimulate the growth and activity of beneficial bacteria in the gut. By using probiotics and prebiotics, farmers can improve the balance and diversity of the gut microbiota of their animals, which can have various positive impacts on their health and performance.

The mechanisms of action by which natural compounds influence glucose metabolism are multifaceted and involve various biological processes. Plant extracts, phytochemicals, and pro/prebiotics interact with various metabolic pathways to regulate glucose levels in the body. For instance, certain plant extracts contain polyphenols and flavonoids that enhance insulin sensitivity and promote glucose uptake by activating insulin receptor signaling pathways. These compounds can also stimulate the activity of glucose transporter proteins (GLUTs), facilitating the transport of glucose into cells, where it can be utilized for energy production. Furthermore, the antioxidant and anti-inflammatory efficacy of the phytochemicals were not only shown to alleviate the stress of the animals but also helped recover the oxidative stress-induced insulin-dependent glucose metabolism pathways [8,9].

Recent studies have shown that probiotics can influence the glucose metabolism of the host animal via several mechanisms, such as modulating intestinal permeability, inflammatory response, hormone secretion, and bile acid metabolism [10,11]. Moreover, the gut microbiota modifying effects of pro/prebiotics can also increase the production of functional microbial metabolites, such as short-chain fatty acids (SCFAs), which can directly or indirectly affect the glucose metabolism of the host animal. SCFAs, such as acetate, propionate, and butyrate, are the primary end products resulting from the fermentation of dietary fibers and other substrates by gut bacteria. Therefore, SCFAs can act as energy sources for the host animal. However, they can also modulate the expression and activity of various genes and enzymes involved in glucose metabolism, such as glucagon-like peptide-1 (GLP-1), peroxisome proliferator-activated receptor gamma (PPAR-gamma), and AMP-activated protein kinase (AMPK). Furthermore, SCFAs can also regulate the host animal’s appetite and satiety, affecting its feed intake and energy balance [12,13,14].

Various studies support the efficacy of these natural compounds, demonstrating their ability to improve metabolic markers and overall health outcomes in livestock. By understanding the biological mechanisms through which these natural materials exert their effects, researchers can better harness their potential as feed additives. This knowledge paves the way for developing targeted nutritional strategies that not only enhance the metabolic efficiency of livestock but also promote sustainable and health-conscious agricultural practices.

Recent research has provided a wealth of information on the potential of natural materials to modulate glucose metabolism in livestock. This review synthesizes these findings, offering a comprehensive overview of the latest studies and their implications. This review also highlights the major challenges related to glucose metabolism in livestock. Moreover, this research underscores the effect of stress on animal glucose metabolism. On the other hand, researchers have identified numerous plant extracts, such as those derived from turmeric, grape, and herbs, which show promising effects on glucose regulation and metabolic health. Additionally, specific phytochemicals, including curcumin, resveratrol, and rosmarinic acid, have been extensively studied for their insulin-sensitizing and blood glucose-lowering properties [15,16,17]. Probiotics and prebiotics have also been highlighted for their ability to modulate animal glucose metabolism by improving gut health and glucose metabolism [18]. By compiling and analyzing these diverse studies, this review aims to pinpoint the most effective natural materials for potential development as feed additives, offering practical insights for enhancing livestock nutrition and metabolic efficiency. The primary goals of this review are to contribute significantly to the advancement of agricultural science and to foster the development of innovative, health-promoting feed strategies. By thoroughly examining natural materials that influence glucose metabolism, this review seeks to offer valuable knowledge that can lead to the creation of more sustainable and effective feed additives. Such advancements are crucial for improving livestock health and productivity, thereby supporting sustainable agriculture’s broader objectives. Ultimately, this review aspires to bridge the gap between research and practical application, encouraging the adoption of natural, science-backed solutions in livestock nutrition that benefit both the animals and the environment.

## 2. Insulin-Dependent Glucose Metabolism Pathways

**Figure 1:**
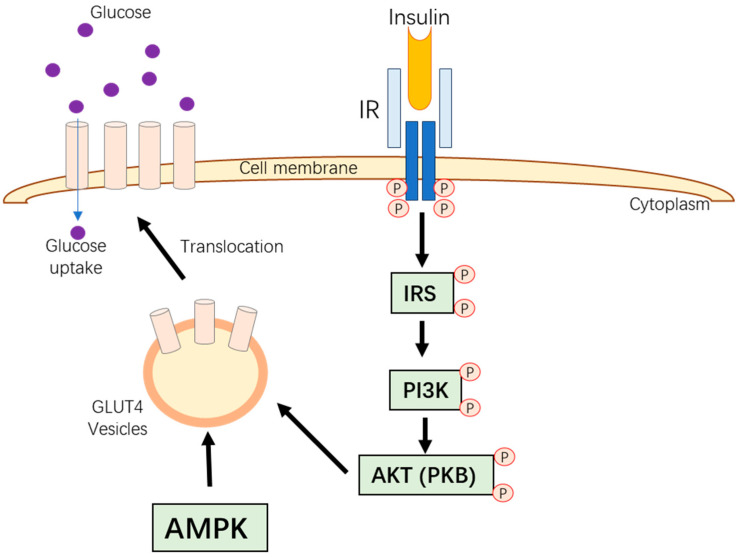
Pathway of insulin-dependent glucose uptake and AMPK activation-induced translocation of GLUT4 vesicles. Pointed arrows represent activation or translocation in the signaling pathways. The “P” symbol with a circle represents a phosphorylation event. After insulin binds to the IR and triggers tyrosine autophosphorylation, the IRSs are activated to promote the PI3K/AKT pathway, eventually inducing the translocation of GLUT4 vesicles to the cell membranes and facilitating glucose uptake.

Insulin is a critical hormone in glucose metabolism, facilitating glucose uptake from the bloodstream into cells, which is vital for energy production and glycogen storage. This process begins with insulin binding to its receptor (IR), triggering intracellular events. The intracellular activities include autophosphorylation of the receptor’s tyrosine residues and insulin receptor substrates (IRSs) activation. This, in turn, activates the phosphatidylinositol 3-kinase (PI3K) and protein kinase B (Akt) pathway, resulting in the movement of glucose transporter type 4 (GLUT4) to the cell membrane, where it facilitates glucose uptake. The PI3K/Akt pathway is thus a key driver in insulin’s metabolic actions (Figure 1) [19].

Glucose transporters (GLUTs) are essential in glucose movement across cell membranes. Different GLUTs are expressed in various cell types; for example, GLUT1 is found in astrocytes and the blood–brain barrier [20], while GLUT4 is insulin-dependent and predominantly found in muscle and liver tissues [21]. However, poultry lack GLUT4, relying on other GLUT isoforms like GLUT1, GLUT3, and GLUT8 in skeletal muscles. These alternative isoforms may perform similar roles to GLUT4. AMP-activated protein kinase (AMPK) is another crucial regulator of glucose metabolism. It is activated during low-energy states by an increased AMP/ATP ratio. AMPK promotes the translocation of GLUT4 to the cell membrane, enhancing glucose uptake [22]. It is also a therapeutic target for managing metabolic disorders like diabetes and obesity.

Stress, both oxidative and metabolic, is a significant factor that negatively impacts glucose metabolism in animals. Oxidative stress arises from an imbalance between reactive oxygen species (ROS) and antioxidants, damaging cells and disrupting insulin signaling [23,24,25]. Metabolic stress, often linked to improper diet formulation or high energy demands, can impair insulin sensitivity and GLUT expression, leading to insulin resistance [26], where cells become less responsive to insulin, thus elevating blood glucose levels. Recognizing and managing these stress factors are crucial in maintaining animal health and performance.

Several strategies can be employed to counter these adverse effects. Improving antioxidant defenses through dietary supplementation with compounds such as phytochemicals, chromium, and selenium can help mitigate oxidative stress. Managing environmental factors such as temperature and housing conditions can reduce metabolic stress. Furthermore, nutritional interventions including the use of phytochemicals, probiotics, prebiotics, or symbiotics in a balanced diet have been shown to improve gut health and support glucose metabolism.

## 3. Major Challenges Related to Glucose Metabolism in Animal Production

### 3.1. Poultry

Glucose metabolism in poultry is essential for optimizing their growth, health, and productivity. Studies demonstrated that chickens have nearly twice the fasting blood glucose levels compared to mammals, while their blood insulin level remains similar to mammals [27,28]. In fact, in chicken skeletal muscles, the mRNA and protein expression levels of the p85 subunit and IRS-1 substrate protein were higher compared to rats [29,30]. Moreover, the chicken tyrosine phosphorylation levels of IR and IRS-1 are twice those observed in rats under basic substrate energy metabolism conditions. Furthermore, chickens have 30 times higher PI3K activity than rats [29,30], indicating higher intrinsic insulin resistance compared to mammals. Another difference in poultry is that, unlike mammals, birds do not express GLUT4; instead, they rely on other glucose transporters such as GLUT1, GLUT3, and GLUT8. GLUT1 is widely expressed in various tissues and is insulin-responsive in chicken myoblasts [31].

Sung et al. [32] highlight the role of glucose metabolism in the growth rate differences among poultry breeds. Arbor Acres broilers, known for their rapid growth, exhibit lower fasting blood glucose concentrations and slower glucose clearance rates than silky chickens, a traditional Chinese breed with a slower growth rate [33]. The evidence showed that genetic advancements aimed at enhancing growth rates in modern poultry could lead to an increased risk of metabolic disorders [34].

Metabolic diseases such as fatty liver hemorrhagic syndrome (FLHS) are often found in caged and over-conditioned laying hens, which has been proven to be a significant noninfectious cause of mortality [35]. FLHS is linked to impaired glucose metabolism, which leads to decreased egg production and can be fatal. It is primarily caused by insulin resistance induced by high-energy, low-protein diets [36]. Insulin resistance in poultry is characterized by elevated insulin levels post-fasting and high glucose levels, resulting in impaired liver fat and amino acid metabolism due to reduced glucose conversion. One study found that the expression of GLUT1 and GLUT3 in the liver of chickens fed a high-energy, low-protein diet was significantly reduced, indicating decreased blood glucose intake in these birds [36].

Insulin also stimulates the conversion of glucose into glycogen or acetyl-CoA, facilitating fatty acid and amino acid synthesis in the liver, which is an important site related to egg production [37]. Notably, only 25% of the fatty acids required for yolk deposition in laying hens are derived from the diet, with the remaining 75% being produced by the liver (70%) and adipose tissue (5%) [38]. This balance further emphasizes the importance of proper insulin levels for efficient production and health in poultry (Table 1).

### 3.2. Swine

Glucose metabolism is vital for the reproduction health of sows. The survival rate of piglets was shown to be highly relevant to maternal glucose metabolism. Kemp et al. [39] reported that multiparous sows that are less glucose tolerant have more significant piglet mortality during the first seven days of farrowing, even without clinical metabolic disorder evidence, which means no glucose and ketone body acetoacetic acid was detected in the urine. This indicated that minor disturbances in glucose metabolism could negatively affect the piglets’ survival rate. The same study also showed that low maternal blood glucose baseline was related to longer pregnancy duration and heavier pig weights at birth.

During pregnancy, the insulin response and glucose metabolism change rapidly. A study on primiparous Landrace × Large White gilts reported that at the end of pregnancy, the gilts had developed insulin resistance, which became more severe during lactation. This is the adaptive response for sows to reduce the glucose consumption of insulin-responsive tissues, such as muscle and liver [40], in order to provide more glucose to the mammary gland for lactation. In addition, mammary alveolar epithelial cells lack GLUT4 expression [41], and many studies showed that glucose uptake and glucose transport in the mammary gland during lactation are insensitive to insulin regulation. Furthermore, Mosnier et al. [42] found that insulin resistance can further cause reduced feed intake during the peripartum period in lactating primiparous sows. The author showed that 0.26% dietary tryptophan supplementation had no effect on replenishing feed intakes.

Improved glucose tolerance does not always return positive results regarding pork meat quality. An interesting finding about swine glucose tolerance and meat quality was reported by Meyer et al. [43], and the research was conducted on the Poland China and Chester White breeding swine with 90–100 kg body weight. Results of the study showed that longissimus dorsi pork from pigs with high glucose tolerance had a paler color (higher Hunter “L” value) and faster pH dropping rate postmortem, which was found to be positively correlated with higher glycogen content and higher insulin secretion. Glycogen in postmortem pork is catabolized to glucose 6-phosphate through glycogenolysis, which occurs in the sarcoplasm of the muscle fiber [44]. Furthermore, one glycogen moiety generates three ATP molecules and two lactate molecules postmortem during the re-phosphorylation of adenosine diphosphate. Lactate was reported to be the primary cause of postmortem meat acidification, which results in lower meat quality indexes such as decreased water holding capacity, lighter color, and lower pH 45 min postmortem [45]. On the other hand, lower glucose-tolerant pigs with low insulin were reported to have a higher mobilization rate of muscle fats, which may have a negative impact on meat sensory evaluation [46]. Up to now, the relationship between pork meat quality and glucose metabolism, especially at a molecular level, has yet to be investigated (Table 1).

### 3.3. Dairy Ruminants

#### 3.3.1. Dairy Cattle

Dairy cattle require substantial blood glucose levels for milk production and fetal growth. The periparturient period (three weeks around parturition) is a critical challenge for dairy cattle management, as the cattle suffer from various physiological stressors [1,2]. 

During pregnancy, the demand for glucose and fatty acids remains high to support fetal development, often resulting in decreased insulin sensitivity and the onset of insulin resistance. After parturition, the drastic surge in milk production depletes the energy reserves of dairy cows without a corresponding increase in dry matter intake, leading to a negative energy balance (NEB) [47]. Furthermore, the gluconeogenic ability of the liver cannot keep up with the glucose demand [48]; this situation is particularly prevalent in high-yielding cows, which experience severe energy depletion and cannot maintain adequate intake. The response of low glucose and insulin levels in the blood often causes the mobilization of fat reserves and increases the formation of the ketone body [48]. This can further cause fat accumulation in the liver [49], causing fatty liver and inhibiting the detoxifying of ammonia to urea. The abnormal blood circulating ketone level with no clinical signs is defined as subclinical ketosis (SCK) [50]. SCK has been proven to be the most prevalent metabolic disease in cattle during transition periods, caused by the prolonged NEB status in the early lactation period. Furthermore, the glucose metabolizing system of post-parturition cattle promoted the mechanism of insulin-independent glucose uptake that favors the mammary gland to prioritize milk production [51], which results in an insulin resistance status similar to human diabetes [52].

When in an NEB status, the body mobilizes fat reserves, releasing non-esterified fatty acids (NEFAs) into the bloodstream, a key indicator of energy imbalance. Typically, NEFA is mobilized from the triglyceride deposits of adipose tissue and used by the animal body as an energy source or re-esterified by the liver [53]. Optimally, NEFA is oxidized completely as fuel for the liver or partially oxidized to produce a ketone body that is released into blood circulation to provide energy for other tissues. Spare ketone bodies can later be reconverted to store fat triglycerides [54]. However, long-term high NEFA levels inhibit the phosphorylation of serine residues on IRS-1, disrupting the tyrosine phosphorylation of IRS-1 and impairing normal insulin signaling pathways, leading to metabolic dysfunction. Furthermore, NEFAs can activate the proinflammatory transcription factor NFκB in hepatocytes, producing TNF-α and triggering the TLR4/PI3K/AKT metabolic axis. This process decreases insulin sensitivity in calf hepatocytes in vitro.

Comprehending and effectively managing glucose metabolism in dairy cattle is not just a matter of scientific interest but a crucial aspect of maintaining their health, optimizing milk production, and ensuring the well-being of both the cows and their offspring (Table 1).

#### 3.3.2. Dairy Sheep and Goats

In dairy sheep and goats, metabolic challenges during the periparturient period are significant but generally less severe than those faced by dairy cattle, largely due to their greater metabolic adaptability [55]. Like cattle, sheep and goats frequently experience NEB during late gestation and early lactation. This NEB is further exacerbated by the fetus’s high glucose demand, which can reach 30–40 g per day during late gestation [56]. To meet this demand, the liver increases gluconeogenesis; however, when energy intake is insufficient, the liver’s ability to metabolize mobilized fat may be overwhelmed, leading to hepatic lipidosis, a condition similar to fatty liver disease in cattle [56]. Twin-bearing ewes and does are at higher risk of developing pregnancy toxemia due to their reduced capacity to clear ketone bodies, further complicating energy metabolism.

Pregnancy toxemia is a common metabolic disorder in small ruminants, characterized by excessive fat mobilization and elevated blood ketone levels, paralleling the symptoms of ketosis in dairy cattle. Clinical manifestations include lethargy, reduced feed intake, and hypoglycemia [57]. Twin-bearing ewes and high-producing goats are particularly vulnerable, and if left untreated, pregnancy toxemia can lead to severe outcomes, including impaired milk production, compromised welfare, and death.

**Table 1:** Production and health issues related to insulin-dependent glucose metabolism.

Farm Animals	Production and Health Issues Related to Insulin-Dependent Glucose Metabolism	References
Poultry	Higher intrinsic insulin resistance	[29,30]
	2. Metabolic diseases caused by genetically high growth rates and unoptimized diets	[32,34,36]
	3. Insulin modulates fatty metabolism, which relates to egg production	[37,38]
Swine	1. Glucose intolerance in sows inhibits the survival rate of piglets	[39]
	2. High metabolic pressure due to lactation and pregnancy	[40,41,42]
	3. Glucose metabolism influences glycogen and fatty acid deposition and alters meat quality	[43,45]
Dairy cattle (especially for high yield)	1. Periparturient NEB	[47]
	2. Fatty liver disease	[49]
	3. Long-term NEFA exposure-induced inflammation	[54]
	4. Clinical/subclinical ketosis	[50]
Dairy sheep and goat	1. Pregnancy toxemia in twin-bearing ewes and high-producing goats	[56]
	2. Hepatic lipidosis	[56]

Abbreviations: NEB, negative energy balance; NEFA, non-esterified fatty acids.

Sheep and goats also experience heightened oxidative stress and inflammation during the periparturient period [58]. If unaddressed, these conditions can aggravate metabolic disorders and reduce overall performance. A comparative study between primiparous (PRP) and multiparous (MUP) ewes revealed that PRP ewes exhibit higher blood glucose levels alongside elevated markers of oxidative stress and inflammation, such as haptoglobin and paraoxonase [59]. On the other hand, MUP ewes demonstrated better milk production and feed intake, resulting in a more favorable energy balance compared to PRP ewes. These findings highlight the importance of targeted care for PRP ewes to mitigate metabolic stress during these critical periods.

## 4. Effects of Stress on Animal Glucose Metabolism

Hans Selye and Harris first introduced the concept of stress in the 1940s, initially describing it as the sum of animals’ non-specific responses to various stimuli [60]. According to [61], stress includes a wide range of potentially harmful forces acting on animals, which can be categorized as either emotional or physical. Stressors continuously challenge the dynamic equilibrium (homeostasis) of living organisms, which is maintained under complex adverse conditions, as highlighted by several researchers [60,61].

Stress triggers the release of glucocorticoids via the regulation of the hypothalamic–pituitary–adrenal axis [62]. These glucocorticoids are known to diminish the expression of protein kinase B (PKB), impede insulin-mediated phosphorylation, and lower the phosphorylation level of glycogen synthase (GS) [63]. As a result, insulin resistance induced by glucocorticoids is linked to suppressing insulin signaling pathways, such as the PKB/AKT and PI3K/mTOR pathways, in mammals [63]. In avian species, corticosterone (CORT) treatment has been shown to decrease the expression of IRS substrate protein and reduce the activity of PI3K associated with IR [64,65]. Additionally, research by Zhao et al. [31] demonstrated that corticosterone can inhibit insulin’s role in glucose uptake within broiler chicken skeletal muscle tissues.

### Effects of Oxidative Damage on Animal Glucose Metabolism

Oxidative damage is initiated by producing reactive oxygen species (ROS) [66]. Under normal conditions, ROS is a metabolic byproduct of cellular function, supporting cell signal transduction and physiological processes such as immune function, apoptosis, and cell differentiation [66]. Regarding glucose metabolism, basal ROS levels are also essential for insulin secretion. Contreras-Ferrat et al. [67] demonstrated that ROS generation and moderate Ca^2+^ influx after glucose stimulation are necessary for activating ryanodine receptor channels, which then increase intracellular Ca^2+^ and promote insulin secretion.

**Figure 2:**
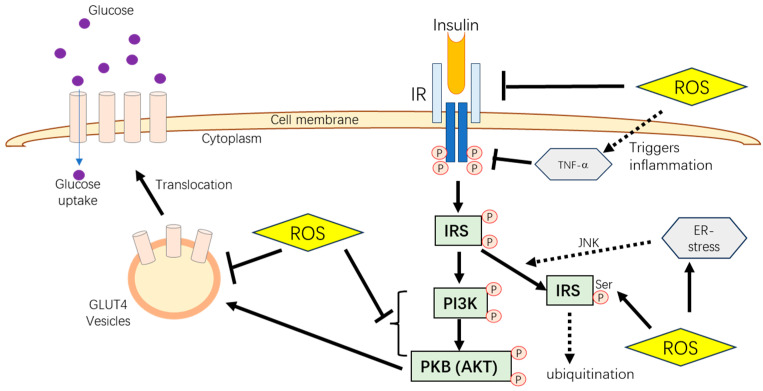
Putative mechanisms by which ROS interferes with insulin-dependent glucose uptake. Pointed arrows represent activation or translocation in the signaling pathways. Line-headed arrows indicate inhibition of the signaling process. The “P” symbol with a circle represents a phosphorylation event. ROS directly inhibits the expression of PI3K/AKB and the translocation of GLUT4. ROS also causes protein misfolding, which can directly inflict IR. The cellular damage can induce the production of TNF-α, which inhibits the phosphorylation of IR. ER stress caused by ROS facilitates the ubiquitination of IRS via the JNK pathway, inhibiting the downstream signaling.

However, specific stressors, such as high-density housing, pathogens, and heat stress, disrupt animals’ oxidative status, suppress growth and production efficiency, and cause health problems. A study of dairy cattle showed that excessive NEFA mobilization during the post-parturient period simultaneously increased ROS production, which could induce oxidative stress and inhibit glucose uptake by interrupting the glucose transporter function and insulin signaling pathway in the liver and peripheral tissues [68]. Furthermore, excess production of ROS can lead to the activation of several serine/threonine kinases, which can further phosphorylate many targets, such as IR and IRS proteins. As a result, increased serine phosphorylation of IRS-1 reduces insulin-stimulated threonine phosphorylation, leading to insulin resistance and type 2 diabetes (T2D) development (Figure 2) [69].

Oxidative stress-induced protein misfolding and the breaking of disulfide bonds have been proven to further cause the onset of stress in the endoplasmic reticulum (ER) [70]. The occurrence of ER stress is a common phenomenon in T2D patients and has been observed in adipocytes and liver cells of genetically obese and high-fat-fed mice models [71,72]. According to the studies, ER stress activates c-Jun N-terminal kinase (JNK). It induces serine phosphorylation of IRS, leading IRS to undergo ubiquitination, eventually inhibiting the insulin-dependent glucose uptake (Figure 2) [73].

Excessive ROS is metabolized by cellular enzymes such as superoxide dismutase (SOD), glutathione peroxidase (GPx), catalase (CAT), and non-enzymatic antioxidants such as vitamin E, β-carotene, ascorbic acid, glutathione (GSH), and exogenously supplied polyphenols [74]. SOD, which depends on trace minerals, is crucial in detoxifying superoxide (O^2−^) in the first step of the enzymatic antioxidant systems. This enzyme catalyzes the dismutation of O^2−^ into oxygen and hydrogen peroxide. CAT then converts H_2_O_2_ into water and oxygen, managing the detoxifying process following SOD activity. Studies indicate a positive correlation between SOD and CAT levels, suggesting that enhancing one often boosts the other. CAT, like SOD, can be elevated by exogenous antioxidant compounds, which improve oxidative status and production performance in animals [75]. Glutathione (GSH) is the most abundant non-protein thiol in avian and mammalian cells, playing a crucial role in redox balance and signaling pathways [76]. In poultry, GSH biosynthesis involves L-cysteine, L-glutamate, and glycine and the rate-limiting enzyme glutamate cysteine ligase (GCL), regulated by Nrf2 activation [77]. The GSH system includes GPx, peroxiredoxins (Prx)-isoforms, some glutathione reductase (GR), and GST. In the glutathione disulfide (GSSG) system, glutathione (GSH) acts as a redox buffer, maintaining the redox status by modifying protein cysteine residues or scavenging H_2_O_2_ [78], while GR catalyzes the reduction of GSSG to GSH. The GSH:GSSG ratio indicates cellular redox balance. Heat stress reduces this ratio in broilers, indicating suppressed redox systems [79].

Oxidative stress occurs when free radical formation exceeds the cell’s ability to eliminate them. Excessive ROS causes lipid peroxidation, damaging cell membranes and lipoproteins. This results in toxic byproducts such as malondialdehyde (MDA) and conjugated diene compounds, which serve as indicators of oxidative status. Lipid peroxidation is a radical chain reaction that rapidly spreads, oxidizing other lipid molecules. Proteins can also be damaged by oxidative stress, leading to structural changes that impair enzymatic activity [70].

Protein and cell membrane damages due to oxidative stress often lead to inflammation by activating monocytes and macrophages, promoting inflammatory responses associated with insulin resistance and diabetes mellitus [80,81]. This stress triggers the pro-inflammatory NFκB pathway and increases the expression of pro-inflammatory cytokines, such as TNF-α [81,82]. TNF-α is known to inhibit the insulin transduction pathway and disrupt glucose metabolism by decreasing the tyrosine kinase activity of the IR [83] (Figure 2). Therefore, inflammation caused by free radicals is another potential link between oxidative stress and insulin resistance.

## 5. Glucose-Modifying Materials as Potential Feed Additives

### 5.1. Phytochemicals

**Figure 3:**
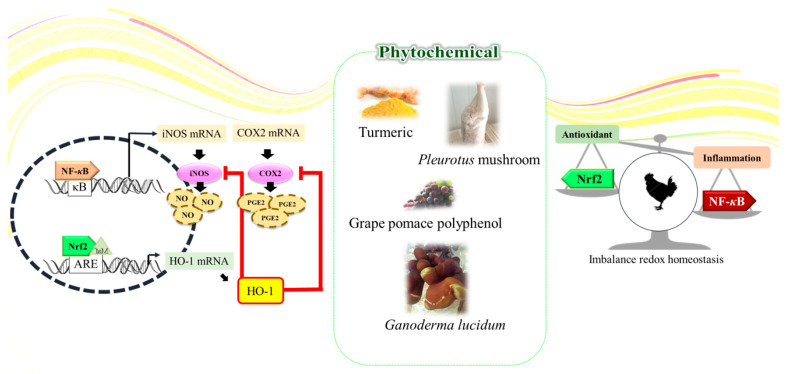
Potential crosstalk between oxidative stress and inflammation through phytochemicals in poultry [75]. Copyright@2019, Animal Bioscience, Seoul, Republic of Korea.

**Figure 4:**
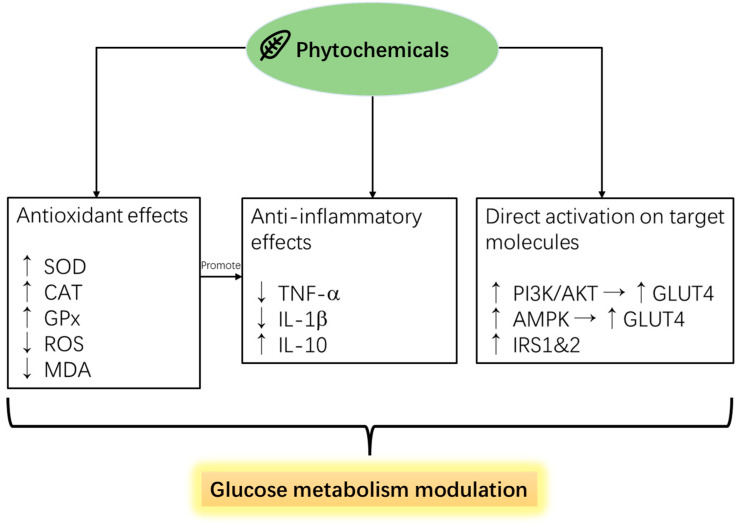
Mechanisms of phytochemicals to modulate glucose metabolism. Upward arrows (↑) and downward arrows (↓) represent the upregulation and downregulation of specific molecules, respectively. The antioxidant and anti-inflammatory effects of phytochemicals prevent the potential inhibition of insulin-dependent metabolism by oxidative stresses. Also, phytochemicals can directly stimulate the expressions of IRSs, PI3K/AKT, and AMPK, eventually facilitating GLUT4 translocation.

Phytochemicals are compounds extracted/derived from plant sources, usually secondary metabolites such as phenolics, triterpenoids, flavonoids, or polysaccharides. Phytochemicals have been well-studied for their medicinal effects on human disease [84] and alleviation effects against stress-induced negative performances in farm animals [85]. Lee et al. previously demonstrated in poultry that phytochemicals’ biofunction has been reported to be linked to their anti-inflammatory and antioxidative effects (Figure 3) [75]. The antioxidant, anti-inflammatory, and potential growth-promoting effects of plant polyphenol compounds have been widely researched due to the trending worldwide regulation of antibiotic growth promoters [86,87]. However, few studies have investigated the relationship between the antioxidant effects and the glucose metabolism-regulating effects of phytochemicals on farm animals. As the previous part mentioned, there are positive links between oxidative stress and inhibited glucose metabolism pathways; reviewing popular phytochemical feed additives with antioxidant efficacies and their effects on diabetic animal/cell models may provide a valuable reference for future studies (Figure 4).

#### 5.1.1. Polyphenols

Phenolics and flavonoids are polyphenols structurally composed of benzoic rings with one or more hydroxyl groups. These two compounds are known for their antioxidant properties due to the highly reducing effects exerted by the hydroxyl groups.

##### Gallic Acid

Gallic acid (GA), a naturally occurring polyphenol, has demonstrated promising potential as a functional feed additive in animal nutrition. Dietary supplementation with GA has been reported to enhance growth performance and overall systemic health in broiler chickens and ruminants. Studies indicate that GA improves feed efficiency, immune function, and oxidative status, suggesting its value in supporting the health and productivity of farm animals [88,89,90]. Additionally, GA has been shown to positively impact gut health by reducing diarrhea in weaned piglets, which is a critical concern in post-weaning management. Supplementing piglet diets with 400 mg/kg of GA significantly reduced diarrhea incidence and improved growth performance, particularly in piglets with lower weaning weights [91].

Beyond its impact on growth and gut health, GA has been linked to enhanced glucose metabolism, particularly through its role in modulating insulin sensitivity. Research on 3T3-L1 cells has shown that GA induces GLUT4 translocation to the cell membrane, promoting glucose uptake [92]. Additionally, GA has been found to activate the AMPK/Sirtuin 1 (Sirt1)/PGC-1α pathway, which is crucial for regulating energy metabolism and thermogenesis in adipose tissue. This pathway’s activation by GA suggests its potential as a therapeutic agent for insulin resistance and metabolic disorders [93].

Furthermore, GA enhances peroxisome proliferator-activated receptor γ (PPARγ) expression in adipose tissue, improving insulin-dependent glucose transport via the PI3K/p-Akt signaling pathway. This mechanism boosts glucose uptake, supports adipogenesis, and protects β-cells from damage, making GA a promising candidate for managing obesity-associated T2DM [94]. These findings underscore the potential of GA as a functional feed additive, improving both animal production and metabolic health by enhancing glucose metabolism and insulin sensitivity.

##### Olive Pomace

Olive pomace (OP), a by-product of olive oil processing, consists of olive skins, pulp, seeds, and residual oil. It is generated at approximately 0.5–0.6 tons per ton of olives processed. One of OP’s most notable attributes is its rich content of functional compounds, particularly polyphenols such as hydroxytyrosol, tyrosol derivatives, and phenolic acids [95]. These polyphenols have been widely studied for their involvement in various biological processes, with potential health benefits for both humans and animals. The presence of these bioactive compounds enhances the value of OP, making it an attractive option for use as a functional feed additive [96].

Given these attributes, OP holds promise as a functional feed additive, offering both nutritional benefits and the ability to modulate glucose metabolism. This dual function could improve animal production and health by enhancing insulin sensitivity and reducing metabolic inflammation. Studies have shown that OP positively affects production and product quality in poultry [97]. Furthermore, in livestock, replacing up to 15% of corn silage with OP in the diets of lactating cows had no negative impact on milk production, composition, or feed efficiency [98]. Additionally, other livestock studies observed no adverse effects on rumen fermentation, nutrient utilization, growth performance, or carcass traits [99], further supporting OP’s potential as a sustainable and functional feed ingredient.

Research has also highlighted other beneficial effects of OP on glucose metabolism. In a study with obese mice, a diet supplemented with pomace olive oil and its triterpene fraction significantly reduced body weight, insulin resistance, and inflammation while improving vascular function despite continued high caloric intake [100]. These findings suggest that the polyphenolic compounds in OP contribute to better metabolic health, particularly in enhancing insulin sensitivity. Additional evidence comes from in vitro studies, where the aqueous extract of OP modulated glucose metabolism in human intestinal cells, promoting a shift towards glucose conservation. This metabolic change was linked to appetite suppression and reduced pro-inflammatory cytokine IL-8, indicating OP’s potential for managing intestinal inflammation and metabolic disorders [101].

##### Resveratrol

Grape pulps are by-products of wine production, making them a cost-effective and environmentally friendly option as a feed additive. Resveratrol is the major polyphenol compound in grape pomace and is known for its potent antioxidant properties. Several studies on ruminants and monogastric farm animals showed the potential of grape pomace to alleviate oxidative stress and improve the antioxidant enzymes, accompanied by improved product quality and growth/production performance. In a study on heat-stressed broiler models, Zhang et al. [102] reported that resveratrol supplementation at 400 mg/kg in the feed significantly increased muscle tissue’s total antioxidant capacity (T-AOC). Similarly, Zhu et al. [103] demonstrated that the same inclusion ratio of resveratrol (400 mg/kg) in the feed significantly improved hepatic T-AOC in yellow-feathered broilers. In lactating dairy sheep, Alba et al. [104] showed that 2% supplementation of grape residue flour (containing 52 mg/g RSV) in a concentrate led to higher levels of SOD and GPx in the serum on day 15, as well as a reduction in lipid peroxidation. Additionally, the serum and milk T-AOC was greater in resveratrol-supplemented sheep compared to the control group.

In a high-fat diet (HFD) rat model, resveratrol supplementation (60 mg/kg BW/day) was reported to elevate the secretion of GLP-1 and insulin while increasing levels of colonic proglucagon mRNA transcripts, in a swine hypercholesterolemic diet model. A 100 mg/kg/d of resveratrol supplementation improved the liver expression of IRS-1 and phosphorylated-AKT, while GLUT4 and p-AKT protein levels in skeletal muscle were up-regulated [105]. These findings suggest the potential modulating effects of resveratrol on glucose metabolism and oxidative status.

##### Curcumin

Curcumin is turmeric’s primary biofunctional phenolic compound with AMPK and PI3k/AKT pathway activation effects, as shown in several studies using cell models [106,107]. In addition, IRS1 protein expression stimulating effects of curcumin were also found in the liver of fructose-fed rats (orally fed 15, 30, and 60 mg/kg bw of curcumin) along with C2C12 and L6 cell experiments (10–20 µm/L) [108,109]. In research about farm animals, 28 d of curcumin supplementation (200 mg/kg diet) was reported to ameliorate the stress on broilers with high stock density by increasing antioxidant activities (including SOD, CAT, GPx) and reducing pro-inflammatory cytokines TNF-α, IL-2, and IL-6 [110]. Furthermore, Chen et al., (2024) reported that *Eimeria tenella*-infected broilers with 200 mg/kg of curcumin supplementation had lower pro-inflammatory cytokine production with higher serum antioxidant capacity than untreated infected birds [111]. In dairy calves suffering from post-weaning stress, curcumin supplementation (65.1 mg/kg DM) improved CAT and SOD activity with a lowered lipid peroxidation index, while proinflammatory cytokine was also decreased [112].

Curcumin was also reported to help with the intrauterine growth retardation (IUGR) of piglets. Newborn piglets with IUGR have light BW, slow development of body organs, and disrupted immune and metabolism systems. One major indicator of IUGR is hyperinsulinemia [113] and high levels of pro-inflammatory cytokines, similar to human T2D syndrome. Hyperinsulinemia can reduce intracellular levels of IRS1 and IRS2 genes in cell culture models and mouse tissues, causing insulin resistance and subsequent diseases [114]. Curcumin improved the growth of IUGR piglets, lowering insulin levels and attenuating inflammation [115].

##### Rosmarinic Acid

Rosmarinic acid is a phenolic compound found in Lamiaceae (Labiateae) plant species or rosemary extracts and is widely used as an antioxidant compound in the food and feed industry. A study supplemented pluriparous dairy ewe with rosemary extracts found that a level of 1200 mg/ewe/d increased milk production, while the level of NEFA was decreased. Interestingly, there were no effects on milk yield with a 600 mg/ewe/d supplementation level, but the blood NEFA concentration was lower than the 1200 mg/ewe/d group [116]. Similarly, in dairy cattle, 28 g/d rosemary extracts supplementation for 74 days significantly increased the milk yield of high-producing dairy cows, accompanied by elevated SOD activity and lowered MDA concentration in blood [117]. In piglets with weaning stress, 100–400 mg/kg feed supplementation of rosemary extracts linearly increased the BW, average daily gain (ADG), and average daily feed intake (ADFI) and decreased the diarrhea index. The same study also showed that piglets supplemented with rosemary extracts had increased SOD and GPx, with reduced MDA in the serum and liver [118]. In broilers, 750 mg/kg rosemary extract feed supplementation improves growth performance with elevated antioxidative status. At the same time, serum glucose was reported to be significantly reduced, suggesting the potential glucose uptake stimulating effects of rosemary extracts in broilers [119].

Treatment with RA at doses of 120–200 mg/kg for seven days significantly reduced blood sugar levels in T1D rats induced by streptozotocin. It also notably enhanced glucose absorption and insulin responsiveness in a dose-dependent manner in T2D rats. This anti-diabetic effect is attributed to a reduction in the liver’s phosphoenolpyruvate carboxykinase levels and an elevation in the skeletal muscle’s GLUT4 levels [120]. To elucidate the glucose metabolism-modulating effects of rosemary extracts, a study based on cell experiments showed that 5 μg/mL of rosmarinic acid increased muscle glucose uptake and AMPK phosphorylation [121]. Interestingly, the mechanism was PI3K/Akt independent, and no GLUT1 and GLUT4 activation was observed. The author further suggested that rosmarinic acid might be involved in preventing palmitate-induced IRS-1 (ser307) phosphorylation and the decline in plasma membrane GLUT4 levels.

##### Quercetin

Quercetin, a flavonoid from the polyphenol category, is increasingly recognized for its beneficial effects on glucose metabolism and growth performance in farm animals. Studies show quercetin supplementation can improve insulin sensitivity and lower blood glucose levels. For example, in angiotensin II-induced hypertensive rat models, quercetin administered at 50 mg/kg/day for 30 days reduced serum lipid peroxidation levels, increased insulin sensitivity in adipose tissue, and improved lipid profiles, as evidenced via insulin tolerance tests and homeostatic model assessment (HOMA) index improvements [122]. In broiler chickens, supplementation with 0.4 and 0.6 g/kg of quercetin significantly increased mRNA expression of AKT and AMPKα1, resulting in improved meat quality and reduced lipid oxidation via the PI3K/PKB/AMPKα1 signaling pathway [123]. The same study also showed that, compared with the control group, supplementation with 0.4 g/kg of quercetin significantly increased the thigh muscle’s pH 45 min and L* value and decreased the shearing force and drip loss of the thigh muscle. Similarly, in streptozotocin-induced hyperglycemic Arbor Acre broilers, quercetin supplementation alleviated oxidative stress by altering antioxidant enzyme activities, decreasing MDA and nitric oxide levels, and activating genes related to the PI3K/PKB signaling pathway [124]. This resulted in decreased fasting blood glucose and increased fasting insulin levels, demonstrating quercetin’s protective effect against hyperglycemia and oxidative damage. Moreover, in broiler chicken without challenge, quercetin supplementation at concentrations of 200, 400, and 800 ppm significantly upregulated the mRNA expression of intestinal Cu/Zn- SOD1, GSH-Px, and nutrient transporters, including GLUT2 and peptide transporter 1 (PEPT1), enhancing antioxidant status and nutrient absorption [125]. In a peripartum dairy cow study, to prevent microbial quercetin degradation in the rumen, quercetin was administered via a duodenal fistula at 100 mg/kg body weight daily from 20 days before to 20 days after calving. This regimen tended to reduce liver fat content. However, it did not significantly alter liver glycogen, glutathione concentrations, or the expression of genes related to hepatic lipid metabolism and antioxidative status. This study showed the potential liver-protecting effects of quercetin [126]. In pigs under transport stress, pigs with 25 mg/kg of quercetin-supplemented feed showed lower intestinal levels of ROS and MDA, which could potentially modulate the glucose metabolism and prevent the deterioration of postmortem meat quality [127].

#### 5.1.2. Terpene-Based Essential Oils

Terpenoids are major functional compounds in essential oil products, known for their antimicrobial, antioxidant, and anti-inflammatory effects during animal production [128,129]. In cell experiments, Zou et al. [130] found that oregano essential oil decreased ROS and MDA production while improving antioxidant enzyme activities in H_2_O_2_-challenged pig small intestinal epithelial cells (IPEC-J2). Tan et al. [131] reported that oregano oil reduced oxidative stress in multiparous sows during early lactation and late gestation, improving piglet performance. Zhao et al., (2023) [132] showed that 1% dietary supplementation with carvacrol–cinnamaldehyde–thymol (CEO) blend improved performance and gut health in piglets by enhancing the intestinal absorptive area, strengthening barrier integrity, increasing digestive enzyme activity, and reducing intestinal inflammation. Reducing inflammation and enhancing antioxidative status may indirectly stimulate feed glucose uptake.

Animal and cell studies have shown that essential oils modulate glucose metabolism pathways. One study showed that carvacrol, the primary component of oregano oil, promoted GLUT4 membrane translocation and restored PI3K/AKT in both streptozotocin-induced T1DM and db/db T2DM mouse models [133]. Furthermore, cinnamaldehyde replenished GLUT4 expression in the skeletal cell membrane of diabetic rats when orally supplied as *C. zeylanicum* extracts (20 mg/kg BW) for two months [134].

In ruminants, essential oils may not exert obvious glucose metabolism-modulating potential due to rumen microbiota metabolism, as evidenced by changes in rumen fermentation and the microbiota profile after administration [135,136].

### 5.2. Probiotics

**Figure 5:**
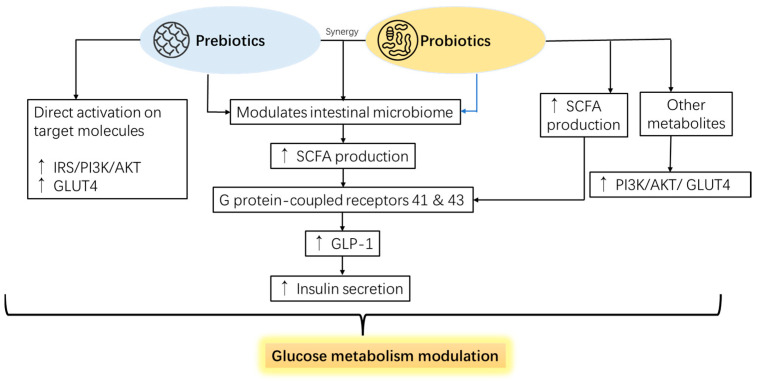
Mechanisms of probiotics and prebiotics on the modulation of insulin-dependent glucose metabolism. Upward arrows (↑) and downward arrows (↓) represent the upregulation and downregulation of specific molecules, respectively. Probiotics and prebiotics can work independently or synergistically to modulate the intestinal microbiome and facilitate the production of SCFAs. The SCFAs then stimulate the GPR 41 and 43, promote pro-insulin GLP-1, and enhance host insulin secretion. Individually, prebiotics can directly promote the IRS/PI3K/AKT pathway and stimulate GLUT4 translocation. On the other hand, probiotics directly stimulate PI3K/AKT/GLUT4 with their metabolites, such as surfactin. Furthermore, some probiotics, after successful colonization in the intestinal environment, can exert their benefits through SCFA production.

Probiotics are live microorganisms that benefit their host when consumed in adequate amounts. Probiotic feed additives improve intestinal health by modulating intestinal microbiomes, essential in overall animal health, including glucose metabolism. Mechanisms of probiotics for their general health-promoting effects have been reported to be (1) competing with the adhesion sites of the pathogens, (2) enhancing the intestinal mucosal barrier, (3) immunomodulation of the intestine, and (4) synthesis of neurotransmitters [137]. Scientists have widely studied the glucose metabolism-modulating effects of probiotics on the anti-diabetic topic and suggested that the mechanism is based on the modulation of microbiota, with the metabolites of the probiotics that directly stimulate the related molecular pathways (Figure 5).

#### 5.2.1. Lactic Acid Bacteria

Lactic acid bacteria (LAB), a group of non-spore-forming, gram-positive bacteria, are widely known for promoting intestinal health. Their role in producing lactic acid and influencing the intestinal environment is crucial. The LABs commonly used in animal feed or food production, including *Lactobacillus*, *Enterococcus*, *Streptococcus*, *Lactococcus*, *Leuconostoc*, *Weissella*, and *Pediococcus*, are of particular importance [138].

A primary mechanism of LAB that modulates glucose metabolism and health in farm animals is its SCFA-promoting effects. SCFAs, especially butyrate, are involved in inhibiting the NFκB pathway, which was reported to further downregulate pro-inflammatory cytokines expression in broilers, including IL-6 and TNF-α [139]. Moreover, studies have demonstrated that SCFAs can inhibit common pathogens are harmful to animal health and performance, such as *Salmonella* spp., *Escherichia coli*, and *Shigella* spp. [140,141]. Nursery pigs supplemented with 10% *L. plantarum* and *P. acidilactici* co-fermented feed had improved weight gain and fecal SCFA concentration due to the improvement of SCFA-producing bacteria in intestinal microbiota [142]. Furthermore, feeding piglets with fermented wheat from *L. reuteri* could enhance the production of SCFAs, leading to improved intestinal health and reduced diarrhea incidence [143].

Regarding glucose metabolism modulation, *L. kefiranofaciens* M and *L. kefir* K supplementation (10^8^ CFU/d) have the effect of stimulating GLP-1 and insulin secretion and elevating the production of anti-inflammatory cytokine IL-10 in the STZ-induced T1D mouse, which is a result of the elevated SCFA production in the intestine. As suggested by the author, increased SCFA activates G protein-coupled receptors, GPR 43 and GPR 41, on enteroendocrine L cells and triggers the production of GLP-1 [144]. Wang et al. [145] reported that *L. reuteri* I5007 treatment improved the PI3K-Akt pathway in the ileum mucosa of piglets, with downregulation of inflammatory cytokines. Although the study did not investigate the glucose transporter actions, the stimulation of PI3K/Akt showed the potential impact of *L. reuteri* I5007 on glucose modulation.

#### 5.2.2. *Clostridium butyricum*

*Clostridium butyricum* (*C. butyricum*) is a Gram-positive obligate anaerobic *Bacillus*. It was first isolated from pig intestines in 1880 by Prazmowski. This bacterium is a common gut commensal in humans and animals and can be found in soils and healthy intestines [146,147].

In animal production, *C. butyricum* has been proven to improve intestinal barrier function, microbiota, and antioxidant status, potentially improving growth performance and animal health [148,149]. Moreover, *C. butyricum* supplementation was reported to attenuate oxidative stress, inflammation, and hepatic fatty deposition in corticosterone-induced liver injury model Pekin ducks, with up-regulation of the PI3k/AKT pathway [150].

The major glucose metabolism-modifying mechanism is the activation of GPR 41 and GPR 43 by butyrate, which stimulates the secretion of GLP-1 from the L cells within the intestinal epithelium [151]. Jia et al. [151] reported *C. butyricum* CGMCC0313.1 increased GLP-1 secretion and resulted in significant improvement of IRS/AKT expression in mice fed high-fat diets, which is in line with increased glucose uptake by insulin-targeted tissues, mediated by glucose transporter GLUT4 and uncoupling protein 1 (UCP1). The same study also mentioned that SCFA receptors Ffar2 and Ffar3 were involved in the mechanism. Therefore, *C. butyricum*, presumably via butyrate, may modulate glucose metabolism and reduce insulin resistance through GLP-1-induced activation of the IRS-1/AKT pathway.

#### 5.2.3. *Bacillus* sp.

The *Bacillus* genus species effectively produce spores, enabling them to withstand digestion via the intestines. Many studies have confirmed that *Bacillus* sp. had the potential to interfere with glucose metabolism by altering PI3K/AKT signaling pathway intervention. For example, Sun et al. implied that B. natto attenuated the obesity index and improved insulin resistance in a high-fat diet-fed rat model by activating the PI3K/AKT signaling pathway [152]. Moreover, Wang et al. [153] reported that *B. subtilis* strains isolated from the ruminal fluid of Holstein dairy cows modulate the PIK3CB–AKT–mTORC1 pathway. 

Furthermore, *Bacillus* sp. metabolites also showed remarkable effects against disturbances in glucose metabolism. For example, surfactin, derived from *B. amyloliquefaciens*, attenuated insulin resistance and enhanced glucose uptake in insulin-resistant HepG2 cells using GLUT4 translocation and influencing PI3K/AKT pathway activation. Remarkably, surfactin evidenced its efficacy in mitigating metabolic disorders by reducing body weight, food intake, and fasting blood glucose in T2D mice [154].

Another prominent example is Hawaijar, a traditionally Indian fermented soybean-based food in which the tradition of fermentation involves mainly microorganisms belonging to the species *Bacillus*. In this study, a protein isolated through purification steps from Hawaijar (ISP, ~24KDa) was seen to induce the PI3K/AKT/GLUT4 pathway in high glucose-treated cells. Notably, ISP-insulin-treated groups significantly raised the PI3K/AKT/GLUT4 pathway and glucose uptake more than either insulin or ISP alone, demonstrating the promoting effects of ISP on the insulin-dependent glucose metabolism pathway [155].

The composition of microorganisms in probiotics is pivotal in determining their efficacy. Multi-strain probiotics have been shown to exhibit synergistic effects, providing greater benefits compared to single-strain formulations [156]. Additionally, different strains, even within the same species, can exert distinct effects on the host’s gut health, nutrient absorption, and immune modulation. For instance, specific strains of Lactobacillus, such as *L. rhamnosus* GG and *L. acidophilus*, have demonstrated therapeutic effects in managing clinical diabetes [157,158]. The survivability of probiotic strains is also a critical consideration. Microorganisms used in probiotics must withstand the harsh conditions of the digestive tract, including the stomach’s acidic environment and exposure to bile salts while competing with the native gut microbiota. In animal feed applications, it is crucial to select strains that can survive the feed processing conditions, such as high temperatures during pelleting, to maintain their viability post-ingestion. *Bacillus* species are excellent candidates in this regard due to their spore-forming properties. For example, *B. subtilis* PB6 has been shown to endure high-temperature pelleting and still exert health-promoting effects in *E. coli*-challenged broilers [159]. Furthermore, the composition of probiotics must be tailored to the host’s specific intestinal microbiota. The gut microbial communities of different species, such as poultry, swine, and ruminants, differ significantly, requiring the selection of probiotic strains that are compatible with each species’ unique gut environment [160]. Based on previous studies, it is feasible to identify probiotic compositions that enhance glucose metabolism and benefit animal production. However, the true impact of these probiotics on production performance should be validated through controlled animal trials.

### 5.3. Prebiotics

Prebiotics are major nondigestible carbohydrates (majorly polysaccharides or oligosaccharides) that cannot be digested by animal digestive enzymes but can be degraded by microbes [161]. Therefore, prebiotics often have no nutritive value, but they have been proven beneficial to animal performance [162]. Common prebiotics used in animal productions, such as mannan oligosaccharides (MOS), xylo-oligosaccharides (XOS), fructo-oligosaccharides (FOS), and beta-glucan, are being utilized for the modulation of gut microbiota, and microbial metabolites, such as SCFAs, play a crucial role in improving animal health [163].

A study showed that mice with 1% MOS in drinking water improved muscle strength, indicating positive effects on muscle growth. The study identified decanoic acid (DA) as the functional metabolite from MOS, which further showed that DA improved the differentiation of C2C12 muscle cells via the PI3K/AKT signaling pathways [164]. Another study demonstrated that MOS from cassia seed gum (300–1200 mg/kg BW/d) improved glucose tolerance and hepatic glucose metabolism of a STZ plus high-fat and high-sugar diet mice model via regulation of the AKT/IRS/AMPK signaling pathway, accompanied by ameliorated intestinal inflammation, and enhanced intestinal integrity [165]. MOS has been shown to exert critical protective effects on intestinal health and performance in pig breeding. Saeed et al. [166] stated that MOS has dual effects on absorbing intestinal pathogens and regulating piglet immune responses. In dairy cattle, MOS supplementation (32 g/d) was shown to increase the milk fat percentage without a significant increment in milk production [167]. A functional food study with an HFD mice model showed that sucrose-free hawthorn leather formulated with 75% FOS and 25% XOS decreased blood glucose and serum lipids, attenuated inflammation in the liver, and improved PI3K, p-AKT, and p-mTOR expression in the liver [168]. In addition, research suggests that β-glucan supplementation significantly reduced GSK-3 (glycogen synthase kinase-3) transcription while significantly increasing insulin receptor, IRS1, PI3K, AKT, eNOS, and GLUT4 activity [169]. Erdem and Roep [170] demonstrated that cereal β-glucan stimulates PI3K/GLUT4 to reduce blood glucose levels by increasing the phosphorylation of insulin receptors, IRS1, and p85-PI3K of T2D skeletal muscles. Additionally, western blot data indicates that cereal polysaccharides enhance the protein activity of IRS2, PI3K, and glycogen synthase in the livers of patients with T2D. This is accompanied by a reduction in the expression of GSK-3, suggesting that cereal polysaccharides (specifically β-glucan) promote glucose activity by activating the PI3K/GLUT4/GSK-3 metabolic pathway [171].

Other prebiotics that are less commonly used in animal production but have been widely utilized for human supplementation also positively impact glucose metabolism regulation. For example, oral administration of inulin (3.33 g/kg bw/days and 1.67 g/kg bw/days) was reported to increase the phosphorylation of AKT in liver tissue and adipose tissue, which increased the expression of GLUT4 and improved glucose tolerance in HFD mice [172]. Interestingly, raffinose, usually seen as an anti-nutritional compound, enhanced GLUT4 translocation via phosphorylation of IRβ/PI3K/Akt in differentiated L6 myocytes and 3T3-L1 preadipocytes [173]. Agriophyllum oligosaccharides, derived from the Mongolian medicinal plant *Agriophyllum squarrosum*, were shown to increase the expression of INS-R, IRS-1, IRS-2, and GLUT4 on the pancreatic tissue of a T2D mice model when orally administered 380 or 750 mg/kg body weight [174]. The mode of action of prebiotics modifying animal glucose metabolism is mentioned in Figure 5.

### 5.4. Symbiotics

Symbiotics is the combination of probiotics and prebiotics. By choosing the adequate fermentation substrate combined with suitable probiotic strains, it is possible to maximize the viability and metabolite-producing efficacy of the microorganisms. The positive effects of symbiotics on glucose metabolism have been well documented. In an investigation of pregnant women with gestational diabetes mellitus, it was shown that symbiotics positively improved fasting plasma glucose, fasting serum insulin, and the homoeostatic model assessment for insulin resistance [175]. In addition, Horvath et al. [176] suggested that symbiotics improved the symptoms, biomarkers, and quality of life in patients with T2D, suggesting a potential role in diabetes management. In addition to the stimulation of G-protein coupled receptors and GLP-1 by SCFAs, it was also indicated that the immune-regulation properties of symbiotics played a role in altering abnormal glucose metabolism, such as diabetes mellitus [177].

### 5.5. Selenium and Chromium

Trace minerals, including Zn, Fe, Cu, Mg, and Mn, are components that build coenzymes that are involved in the overall metabolism cycle (gluconeogenesis, glycolysis, pentose phosphate pathways, and tricarboxylic acid cycle) in humans and animals [178]. Most essential trace minerals have been thoroughly considered and included in the feed formula or feed premix in animal feeding practices. However, various reports suggested that supplementing trace minerals on top of regular feed formula improves animal performance and alleviates stress [179,180]. Selenium (Se) and chromium (Cr) have been trending as feed supplements because of their anti-stress and glucose metabolism-modulating properties.

Selenium (Se) is a trace mineral usually used in animal feeds for its antioxidative and reproduction enhancement effects. Accumulating data indicates that Se exhibits insulin-mimetic effects by activating Akt and serine/threonine kinases that initiate IRS phosphorylation and the subsequent insulin signaling cascade [181]. Selenium supplementation has been shown to increase the secretion of GLP-1 and insulin, accompanied by the increased antioxidant status of diabetic mice [182]. Moreover, selenium has been shown to reduce ER stress due to its antioxidant properties [183]. Selenium has been focused on its antioxidant effects in animal production by replenishing selenoprotein GPx and its potential anti-inflammatory effects [184]. It is worth noting that anti-inflammation effects can improve glucose metabolism since the major inflammatory cytokine TNF-α was reported as the primary factor interfering with the insulin signaling process [185]. A study on glucose tolerance in dairy cattle showed that dietary inclusion of 3.5 mg organic selenium/kg DM concentrate plus 2000 IU vitamin E/day before calving improved insulin sensitivity during the first week of lactation [186].

Chromium (Cr) has recently been trending as an oxidative stress alleviator and glucose metabolism modulator for farm animals. Cr binds to apo-chromodulin to form holo-chromodulin and connects with tyrosine kinase fragments on IRS 1 and 2 to activate its activity, triggering the insulin signaling cascade, ultimately causing GLUT4 to translocate to the cell membrane, increasing the efficiency of glucose transport and promoting the glucose absorption in feed [187]. Interestingly, studies demonstrated that chromium supplementation did not up-regulate IR phosphorylation levels. Instead, the insulinotropic mechanism of chromium is caused by the tyrosine phosphorylation of IRS-1, which means the mode of action is based on the downstream of the IR. This further elevates the phosphorylation of AKT and increased PI3K activity [188,189]. Moreover, evidence has shown that chromium activates AMPK [190] and protects IRSs from ubiquitination by alleviating ER stress [191].

**Figure 6:**
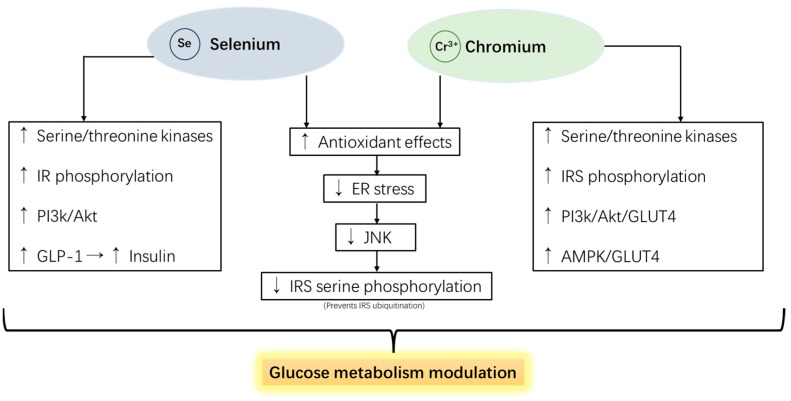
Mechanisms of Se and Cr^3+^ on the modulation of insulin-dependent glucose metabolism. These two trace elements can reduce cellular ER stress via their antioxidant effects. Upward arrows (↑) and downward arrows (↓) represent the upregulation and downregulation of specific molecules, respectively. The reduced ER stress prevents IRSs from ubiquitination, which preserves the downstream signaling cascade of insulin-dependent glucose uptake. Se and Cr^3+^ have been reported to promote the activation of serine and threonine kinases with subsequent IRS phosphorylation. Se and Cr^3+^ have the ability to respectively stimulate GLP-1 for increased insulin production or activate AMPK-facilitated GLUT4 translocation.

The benefits of chromium supplementation have been proven to be the most effective when animals encounter heat or metabolic stress. Hayirli et al. [192] reported that chromium–methionine supplementation attenuated insulin sensitivity prepartum and enhanced glucose tolerance postpartum in dairy cattle. In addition, the same study showed that basal insulin concentrations for cows receiving Cr-Met were higher than for cows not receiving Cr-Met prepartum. In another study on dairy cattle, chromium propionate (CrProp) supplementation resulted in trends for increased DMI and lower plasma NEFA prepartum [193]. Sumner et al. [194] proved the efficacy of glucose metabolism modulating with a glucose tolerance test model on growing Holstein heifer. The study showed that long-term CrProp supplementation increases the glucose clearance rate and results in a higher insulin baseline compared to the non-supplemented control. Studies on swine showed that chromium supplementation increased the inhibited serum insulin concentration due to heat stress and improved the glucose clearance rate in GTT while alleviating stress indices [195]. In poultry, Sahin et al. [196] reported that heat-stressed broilers supplemented with CrPic and CrHis had higher GLUT2 expression in muscle and lower proinflammatory transcriptional factor NFκB, accompanied by replenished growth performance. Mechanisms of chromium and its promotion of animal glucose metabolism are illustrated in Figure 6.

## 6. Limitations for the Feed Additives

Most commonly, phytochemicals, especially plant polyphenols, are made available to farm animals as crude extracts, concentrates, or by-products from the food industry. These forms decrease the cost of feeding additives but come with variable composition and concentration, respectively. However, studies have shown that the amount of phytochemicals in plants can vary due to different harvest seasons, species, and soil conditions [197]. The variability in the composition and concentration in these forms can lead to inconsistent efficacy and require careful formulation to ensure the desired outcomes. Some phytochemicals with higher doses can be pro-oxidative [198], which may put animals into an oxidative stress status and adversely influence animal health and performance. As reported in a study, the oxidation of flavonoids into o- or p-quinones can trigger pro-oxidation due to their highly reactive property towards nucleophilic thiols, amino groups of proteins, and glutathione. Depleting glutathione and modification of proteins collectively enhance oxidative stress within the cell [199]. Furthermore, some flavonoid–redox complexes can cause the production of H_2_O_2_, which leads to the oxidation of hemoglobin (Hb) [200]. The oxidized Hb then releases the Fe+2 ions to undergo auto-oxidation and trigger pro-oxidant effects [201].

As mentioned, probiotics primarily influence animal health by modulating the gut microbiome and producing beneficial metabolites like butyrate. This indicates that probiotics’ effects on growth performance and glucose metabolism are mediated indirectly. As a result, the specific influences on animal performance can be variable and dependent on multiple factors, including the age and type of animals, the existing microbiome composition, and the feed formula. For example, the beneficial effects of probiotics are more evident in broilers before 21 d or weaning piglets, indicating that the gut microbiome in young animals, which is still developing, is more easily altered by probiotic feed additives [18]. Moreover, the effects of probiotics may require a significant amount of time to exert beneficial effects since the modification of the microbiome happens gradually. Research reported that long-term probiotic supplementation exerts better efficacy than short-term [202,203]. This slow process requires a more extended period of consistent supplementation before the benefits are observed. Furthermore, several studies have demonstrated that the benefits conferred by probiotic supplementation often diminish after discontinuation. Evidence suggests that when probiotic use ceases, there is a significant tendency for the gut microbiota to revert to its original composition, which can undermine the health advantages observed during supplementation [204]. This phenomenon is primarily attributed to the inherent plasticity and responsiveness of the gut ecosystem, which is continuously shaped by factors such as diet, environment, and host physiology. The transient nature of probiotic colonization means that without ongoing supplementation, the introduced beneficial strains are gradually outcompeted by native gut bacteria, leading to a re-establishment of the pre-existing microbial community.

Some non-starch polysaccharides (NSPs), such as raffinose, are also reported to have prebiotic effects and promote glucose metabolism [173]. However, NSPs can increase the viscosity of intestinal contents and impede nutrient absorption and overall digestive efficiency. Research has shown that raffinose can reduce growth performance in pigs by lowering feed intake and nutrient digestibility while also inducing a humoral immune response.

Chromium and selenium only require a trace amount to have noticeable effects, which raises concerns about overdosing in regular practices. Official FDA regulations specify a maximum of 300 ppb of selenium as a feed additive in complete feed for various animals, including chickens, swine, turkeys, sheep, cattle, and ducks. The permissible forms are selenite and selenium yeast [205]. The FDA regulation on chromium levels in animal feed specifies a maximum of 500 ppb in cattle and 200 ppb in poultry and swine diets [205]. The safety of CrProp for dairy cattle and overdosing risk have been thoroughly evaluated by Loyd et al. [206]. In this study, 4 times the FDA-permitted dose (2 mg/kg DM) was provided to dairy cattle for more than 121 days; results showed that the overdosed CrProp supplementation did not affect Cr concentrations in milk, muscle, or fat. These results eliminated the safety concern of CrProp supplementation for dairy cattle and its derived products. Similarly, a study on the effects of 10 times the FDA-permitted CrProp dose (2 mg/kg feed) on a broiler showed that Cr concentrations in breast muscle or skin with adhering fat had not changed. However, the same study indicated that overdosed CrProp decreased feed intake and gain in male broilers [207]. Interestingly, some past studies showed the toxic and mutagenic effects on animal and cell models exclusively in chromium picolinate [208]. An NIH-commissioned study later clarified this concern, showing that up to 5% of the diet (by mass) of male and female rats and mice for up to 2 years showed no harmful effects on female rats or mice or male mice [209]. Therefore, selenium and chromium supplementations are considered safe. However, careful dosing and ongoing monitoring are suggested to ensure that administration levels remain within safe and beneficial ranges.

## 7. Research Gaps and Future Direction

Natural supplements that modulate glucose uptake and insulin signaling have recently been trending topics in human medical research. Those topics mainly focused on natural therapeutic materials, such as superfoods or supplements that help patients with clinical syndromes related to T1D and T2D. Research on a specific material often includes a cell experiment to validate the mechanism, followed by animal models to evaluate further the material’s overall effect on diabetic syndromes. High-fat-high-sugar-induced T1D models or streptozotocin-induced T2D models in mice and rats are the most commonly used and well-established methods. The natural materials discussed in this review have demonstrated their effects and mechanisms in these rodent models. However, even though farm animals can experience high blood sugar, metabolic syndromes, and insulin resistance similar to clinical diabetes, there has been limited research focused on the mechanism of the modulation of insulin-dependent glucose metabolism in these animals.

The relationship between metabolic stress and the disruption of insulin-related glucose metabolism has been extensively studied in periparturient dairy cattle, with some research also focusing on periparturient sows. In poultry, a study on broiler chicken (without challenge) also showed the improvement of GLUTs, accompanied by elevated antioxidant status, after the supplementation of cornelian cherry extract. Sahin et al. [197] reported that chromium supplements improved heat-stress-suppressed GLUT2 expression in broilers, enhanced antioxidant status, and reduced inflammation. While some studies have explored the impact of oxidative stress on glucose metabolism suppression and its mitigation through functional feed additives, much remains to be discovered. These studies suggest potential crosstalk between metabolic stress, oxidative/inflammatory status, and glucose metabolism, which warrants further exploration in farm animals.

**Figure 7:**
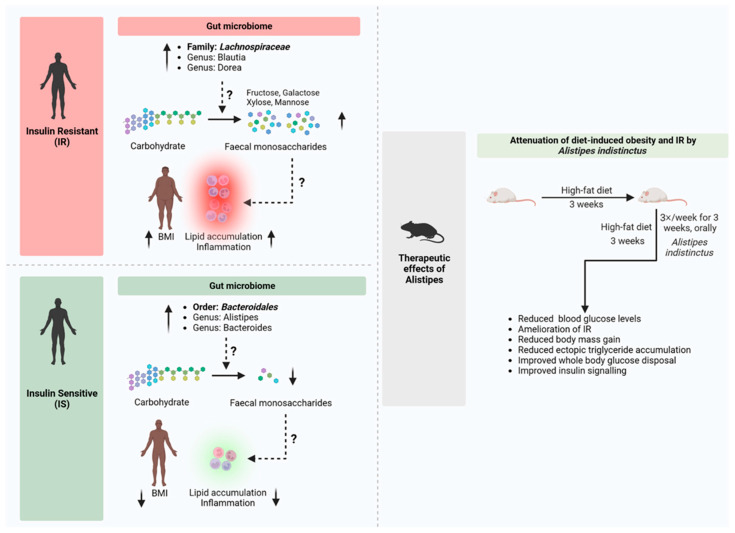
The importance of gut microbial metabolism in regulating insulin sensitivity in humans and mice [210]. Upward arrows (↑) and downward arrows (↓) represent the upregulation and downregulation of specific molecules, respectively. Question mark (?) indicates the hypothesized modulation effects. Copyright@2024, Nature, London, UK.

Moreover, intestinal microbiota and their metabolism largely impacted insulin sensitivity status, as shown in human and mice animal models. It was found that in insulin-resistant individuals, the gut microbiota is enriched with *Blautia* and *Dorea* of the *Lachnospiraceae* family, disrupting carbohydrate metabolism and leading to the accumulation of fecal monosaccharides. Conversely, insulin-sensitive individuals have higher levels of Bacteroidales-type bacteria, such as *Alistipes* and *Bacteroides*. These bacteria’s metabolic activities significantly reduce fecal monosaccharides, resulting in lower lipid accumulation, decreased inflammation, and protection against obesity. Remarkably, administering *Alistipes indistinctus* to mice on a high-fat diet completely prevented insulin resistance and obesity. Therefore, it is possible to target these specific microbes and make them potential probiotic feed additives that improve animal glucose metabolism. Moreover, as mentioned in the same study, fecal monosaccharide levels could be a supportive assessment for evaluating the glucose metabolism status of the host animals (Figure 7) [210]. 

Future research jobs on this topic include investigating the effects of oxidative stress and inflammation that disrupt insulin-dependent glucose uptakes. More specific indicators and indexes in farm animals can be identified to indicate oxidative/inflammatory damage and disrupted glucose uptake. For example, NEFA values have been used in dairy cattle to measure metabolic stress. The RQUIKI (Revised Quantitative Insulin Sensitivity Check Index) has been proven to indicate the insulin function index [211]. In terms of oxidative stress, T-AOC and MDA are used to represent the overall oxidation level in the animal body. At the same time, cytokines such as TNF-α and IL-1β indicate the inflammatory status. By combining the oxidative/inflammation indexes and glucose metabolism indexes, more extensive evaluations can be made regarding academic research or health diagnoses. Furthermore, the underlying molecular pathway and the crosstalk should be the key to evaluating the effect of glucose metabolism-modulating feed additives.

## 8. Conclusions

**Figure 8:**
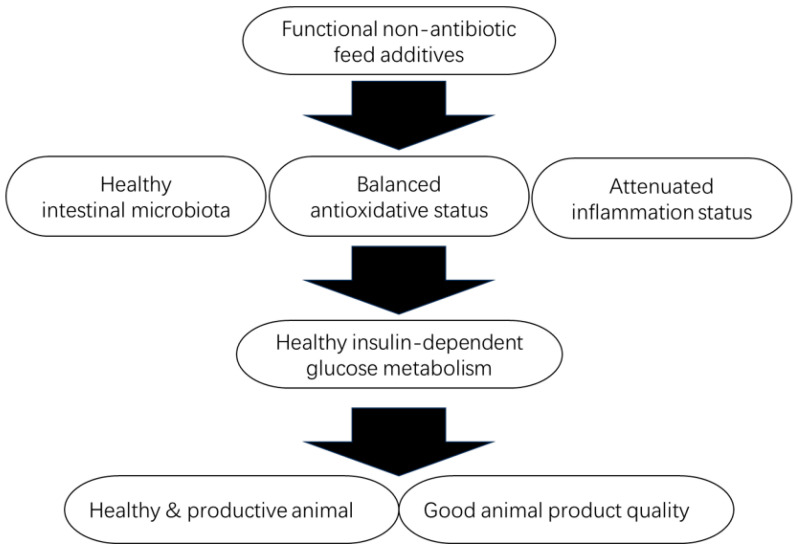
Flow chart showing how functional non-antibiotic feed additives support animal health and preserve good product quality.

From the perspective of current animal farming, glucose metabolism may not yet be a significant topic that everyone focuses on when working on improving the performance of animals. However, the uptake of blood glucose and its utilization in the cells can substantially alter the outcome of animal production. Furthermore, various stress factors during animal production can reduce insulin sensitivity and decrease the metabolizing efficiency of glucose. By reviewing the data from the diabetic animal and cell model, we get to realize the related pathway and the potential modulation mechanism of phytochemicals (Figure 4), probiotics (Figure 5), prebiotics (Figure 5), and selenium and chromium (Figure 6) to prevent stress-suppressed glucose uptake efficiency. Adapting these methods to predict the potential glucose intolerance and inefficiencies of glucose utilization more precisely (e.g., peripartum cattle) while trying to improve glucose metabolism with different feed additives can be a project for farmers and product developers to work on (Figure 8). This approach may also lead to innovative feed additive products in the future.

## Data Availability

No data were created during the preparation of this article.
